# The influence of age on brief motivational intervention for unhealthy alcohol use

**DOI:** 10.1016/j.dadr.2024.100313

**Published:** 2024-12-15

**Authors:** Belina Rodrigues, Nicolas Bertholet, Jean-Bernard Daeppen, Jacques Gaume

**Affiliations:** a Department of Psychiatry – Addiction Medicine, Lausanne University Hospital and University of Lausanne, Lausanne 1011, Switzerland; b Life and Health Sciences Research Institute (ICVS), School of Medicine, University of Minho, Braga, Portugal; c ICVS/3B’s (Life and Health Sciences Research Institute/Institute of Biomaterials, Biodegradables and Biomimetics), PT Government Associate Laboratory, Braga, Guimarães, Portugal

**Keywords:** Brief motivational interviewing, Age, Unhealthy alcohol consumption, Counsellor skills

## Abstract

**Introduction:**

The influence of age on brief motivational interventions (BMI) effects remains unknown. In the present study, we explored whether change in alcohol consumption after BMI differs across age groups and whether these differences are reflected in motivational interviewing (MI) counsellor skills.

**Method:**

Secondary analysis of a randomized controlled trial among emergency room (ER) patients screened for unhealthy alcohol consumption. Participants (N = 97, 80 % men, 18–21 y: 19.6 %, 22–29 y: 22.7 %, 30–49 y: 34.0 % and ≥50 y: 23.7 %) received a single BMI in the ER, which was coded using the MI Skills Code 2.0. Alcohol outcomes were measured at 12-month. First, we tested whether BMI effect varied by age group using negative binomial regression for weekly drinking consumption, and logistic regression for change to low-risk drinking. Second, MI counsellor skills (global ratings of empathy, MI spirit and acceptance, and percentages of open questions, complex reflections (CR) and MI-consistent behaviors) were examined through one-way ANOVA or Welch test.

**Results:**

The 22–29 y group i) reported lower consumption at follow-up compared to the 30–49 y group (IRR=1.60, *p* = .04) and the ≥ 50 y group (IRR=1.67, *p* = .03), and ii) was more likely to change to low-risk drinking than the 18–21 y group (OR=11.25, *p* = .04). When comparing MI counsellor skills across age groups, higher empathy ratings (F(3,93)= 2.70, *p* = .05) and a higher percentage of CR (F(3,93)= 4.10, *p* = .009) were recorded for the 22–29 y group.

**Conclusion:**

This exploratory study shows that BMI was associated with significantly better 12-month alcohol outcomes among patients aged 22–29 years, which corresponded with higher counsellor empathy ratings and percentage of CR.

## Highlights

• The influence of age on brief motivational interventions (BMI) effects remains understudied.
• Findings showed that the 22–29 y group had the best alcohol outcomes.
• Moreover, counsellors’ MI skills during the BMI sessions of the 22–29 y group were higher overall.
• Variations in BMI outcomes may partly stem from patients' age or the interplay between patients' age and counselors' age.
• Understanding the role of age in BMI effectiveness might thus be important for enhancing the impact of interventions.

## 1 Introduction

Motivational interviewing (MI) is an evidence-based treatment for several conditions, including alcohol use disorders (Lundahl et al., 2013), yet MI has yielded results with varying effect sizes (Klimas et al., 2018, Smedslund et al., 2011). Several factors have been put forward to explain this issue. Among these factors, aspects pertaining to the content and type of the intervention are worthy of note. For instance, the MI approach has been adapted to interventions including brief motivational interventions (BMI) and Motivational Enhancement Therapy (Burke, Arkowitz, and Menchola, 2003), and the contribution of the components that differ across these adaptations is unknown. Another aspect is the fidelity to the approach. As many trials do not report fidelity results, it is difficult to ascertain whether studies reporting lower impact are due to the lack of effect of MI or to low fidelity (Miller and Rollnick, 2014). This is not trivial as the level of competence of the counsellor influences the effectiveness of MI. For instance, in a study using the same dataset as the present study, counsellors with better MI skills achieved better results regardless of the patient's ability to change. In contrast, those with lower skills were effective only with patients who had high ability to change (Gaume, Gmel, Faouzi, and Daeppen, 2009).

Mixed results further cloud this issue as to which MI mechanisms are associated with behavior change. Miller and Rose (2009) hypothesized that MI efficacy could be explained by counsellor technical skills, which, when applied, encourage patients to elicit statements favoring change, or so called "change talk" (CT), which would in turn lead to behavior change. However, whereas higher frequency of technical skills is associated with increased CT, it is also associated with increased sustain talk (ST), i.e., statements against change (Magill et al., 2018). Also of concern for this hypothesis, CT often fails to relate with actual behavior change (Magill et al., 2018). On the other hand, the relational hypothesis posits that the relational factors such as empathy and MI spirit are associated with behavior change. However, no significant association was found between relational factors, CT and at-risk behaviors reduction (Magill et al., 2018). Likewise, Bertholet et al. (2014) concluded that counsellor's acceptance, empathy, MI spirit, and patient's self-exploration were not consistent predictors of drinking outcomes. While both hypotheses emphasize counsellor's behaviors, patients' characteristics cannot be discounted as patient's alcohol-related disorder severity was shown to moderate the relationship between MI-consistent behaviors and drinking outcomes (Gaume et al., 2016) and readiness to change has shown to play a role in the effectiveness of BMI (Stein et al., 2009).

Aspects such as the patient’s age could also partially explain the varying sizes effects of MI. In a recent study investigating the differences between younger (<51 y) and older adults (≥51 y) across behavior change conditions (Kuerbis, Behrendt, and Morgenstern, 2023), MI elicited a greater reduction in drinking among older adults than younger adults (Hedge’s g =.44, −12 vs. −3 standard drinks, respectively). However, younger adults reported a greater reduction in drinking compared to older adults four weeks after a session of nondirective listening condition (Hedge’s g =.48). These findings suggest that age might influence the effectiveness of MI. To explain these differences, one could consider whether the mechanisms of change vary throughout life stages. For example, as adolescents have shown positive behavior change in MI even in the absence of ambivalence, Ewing, Apodaca, and Gaume (2016) argued that ambivalence might not be as essential for behavior change among adolescents as it seems to be for other age groups. This implies that the influence of MI components might vary between age groups. Additionally, research on the interaction between patients and health professionals has also pointed to differences between age groups. For instance, Barata, Tokuda and Martins (2012) reported that Portuguese older adults over 60 years old considered attention given by the family doctor as the most important trait in a medical appointment, whereas younger subjects rated more favorably doctor’s competences such as scientific knowledge and ability to solve problems. Altogether, these findings suggest that age should be considered when investigating the effect of BMI on behavioral outcomes.

Several reviews have highlighted that MI has potential to change behavior among children (Gayes and Steele, 2014), adolescents and younger adults (Cushing et al., 2014, Tanner-Smith and Lipsey, 2015), as well as older adults (Purath, Keck, and Fitzgerald, 2014). However, despite the growing number of studies on specific age groups, the heterogeneity among studies precludes the direct comparison of effect sizes, which hinders an evaluation of the influence of age on the effectiveness of MI. To address this issue, we explored in the present study i) whether change in alcohol consumption after BMI aiming at decreasing alcohol consumption among Emergency Room differs across age groups and ii) whether such differences are reflected in counsellors’ MI skills.

## 2 Methods

### 2.1 Study design and sample

**Fig. 1:**
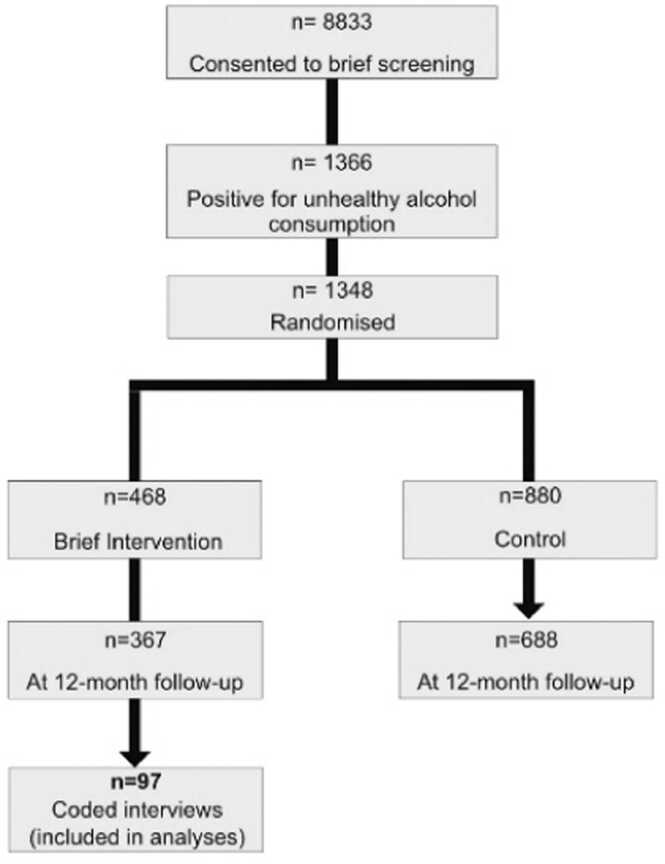
Sample size.

The present study is a secondary analysis of data collected in a randomized controlled trial carried out in the Emergency Room (ER) of Lausanne University Hospital, Lausanne, Switzerland (Daeppen et al., 2007). This trial tested the effectiveness of a single BMI session in reducing unhealthy alcohol consumption at 1-year follow-up. This study received ethics approval from the Ethics Committee of Lausanne University Hospital and was conducted in accordance with the Declaration of Helsinki. Among all patients aged 18 years old and older admitted to the ER after an injury between January 2003 and June 2004, 8833 patients consented to fill in a brief screening for unhealthy alcohol consumption (Fig. 1). This screening was conducted according to the current National Institute on Alcohol Abuse and Alcoholism (NIAAA) standards, i.e., ≥ 14 drinks per week or ≥ 4 drinks on a single occasion in the past 30 days for men aged less than 65, and ≥ 7 drinks per week or ≥ 3 drinks on a single occasion in the past 30 days for men aged 65 or older and women. One drink was defined as containing 10–12 g of pure alcohol. Of the patients who consented to screening, 1366 were positive for unhealthy alcohol consumption. These patients were randomized to one of three groups: screening only control group, screening and assessment control group, and screening, assessment and BMI group. A one-year follow-up was completed by 1055 patients (77.2 %).

The BMI group received a single standardized BMI session lasting 18 minutes on average (SD = 5.9) and including six steps: 1) thank the subjects for participation and reassure about the confidentiality of the session; 2) provide feedback about the individual alcohol use in comparison to Swiss men and women and ask their opinion about the provided feedback; 3) ask the patients to explore the advantages and disadvantages of their alcohol consumption; 4) rate importance and readiness to change consumption on a 10-point scale in order to elicit CT; 5) inquire whether the patient feels ready to set an objective and provide positive reinforcement about individual ability to achieve the defined objective and 6) handout writing material such as the Alcohol Use of Disorders Identification Test (AUDIT) score, the percentile of their drinking pattern and the drinking objectives. Among those included in the BMI group, 166 patients had their session audio-recorded for fidelity monitoring purpose. Of those, 97 were included in the present analysis. The remaining sessions were excluded for the following reasons: 33 lost to follow-up, 25 incomplete data records, seven mismatched identification, three whose French fluency was deemed insufficient and one whose relative interfered during the session. All participants included in the study analysis (n = 97) had complete data with no missing values.

### 2.2 Socio-demographic measures and alcohol outcomes

Sociodemographic data such as gender, age and occupational status (working and not working, i.e., students, unemployed or retired) were collected in the baseline assessment. As for the age groups, we considered “late adolescents” to be aged 18–21 y, “young adults” 22–29 y, “adults” 30–49 y and “older adults” ≥ 50 y. Early old age was considered as ≥ 50 y as Salthouse (2019) found that, with a quasi-longitudinal approach, 50’s were already associated with cognitive decline. The cut-off age between young adults and adults was based on Sawyer et al. (2018) who defined young adults as individuals aged until their late twenties. The cut-off between late adolescents and young adults was defined based on neuroimaging studies which found differing activation patterns in response to CT and ST between late adolescents aged up to 21 y and adults aged ≥ 21 y (Ewing et al., 2013).

We assessed severity of alcohol use disorder at baseline using the French version of the Alcohol Use Disorders Identification Test (AUDIT, Gache et al., 2005). The AUDIT score ranges from 0 to 40, and values from zero to six (for men) and 7 (for women) represent a low risk of alcohol-related disorder, values from 7 (for women) and 6 (for men) to 12 represent an unhealthy consumption and values greater than 12 indicate probable alcohol dependence (Gache et al., 2005).

Alcohol outcomes were measured both at baseline and follow-up. Weekly drinking consumption was assessed by multiplying the frequency of usual drinking by the quantity of usual drinking in standard units of alcohol. Unhealthy alcohol consumption status was calculated based on NIAAA standards, see above.

### 2.3 Tape recording and coding

Two master level psychologists experienced in MI were trained in using Motivational Interviewing Skills Code (MISC) 2.0 (Miller et al., 2003). Briefly, the training was divided into two parts. Firstly, the two raters coded training sessions together. Then, they did it individually. Both parts were supervised and discrepancies between the two coders were discussed and solved by a third rater, who had expertise in MI. This training lasted until sufficient inter-rater reliability was obtained. The two coders then coded each BMI session while blinded to baseline and follow-up data. All 97 sessions were coded by the two coders and the frequencies of each code were averaged across the coders.

MISC coding process is divided into two steps. First, the coders listened to the complete audio tape in order to assess global ratings. For counsellors, acceptance, empathy, and MI spirit were evaluated on a 7-point Likert scale where 1 corresponded to the lowest score and 7 to highest score. The coders then listened to the audio tape a second time, to parse and attribute code to every utterance of the patient and the counsellor. Briefly, counsellors’ utterances included MI-consistent behaviors (MICO, i.e., advise with permission, affirm, emphasize control, open question, simple and complex reflections, reframe, and support), MI-inconsistent behaviors (MIIN, i.e., advise without permission, confront, direct, raise concern without permission and warn) and Others (i.e., “neutral” codes such as filler, facilitate, giving information, closed question, and structure). Patients’ utterances were also coded but are not used in the present analysis.

Several summary scores can then be computed using the MISC (Miller et al., 2003). In the present analysis, we used the following: percentage of MICO (MICO/(MICO+MIIN)* 100), percentage of complex reflections (complex reflections/(simple+complex reflections)* 100), and percentage of open questions (open questions/(closed+open questions)* 100).

The reliability between the two coders was ascertained through intra-class correlations (ICC) for each individual counsellor’s and client’s code and was fair to excellent overall (Gaume et al. 2008a; Gaume et al., 2008b). For measures retained in the present analysis, ICC was 0.53 for acceptance, 0.50 for empathy, 0.53 for MI spirit, 0.83 for percentage of MICO, 0.56 for percentage of complex reflections, 0.82 for percentage of open questions.

### 2.4 Data analysis plan

Analyses were conducted using SPSS version 24.0 (SPSS Inc. Chicago, IL, USA). We first described variables overall and by participant age group using standard descriptive analyses (One-Way ANOVA for continuous variables and *X*^2^ test for categorical variables).

To study the influence of participant age group on change in alcohol outcomes after the BMI, we entered participant age groups as dummy coded variables in regression models for each outcome. For weekly drinking consumption, we computed a negative binomial regression predicting weekly drinking consumption at follow-up, controlling for weekly drinking consumption at baseline. To examine whether there was a change to low-risk drinking by participant age group, a logistic regression was carried out. The models were tested first with participant age groups only, and then adjusted for gender and AUDIT score at baseline. Results were similar in the adjusted and non-adjusted models; we present only the adjusted models below.

We finally compared MI counsellor skills by participant age group. MISC summary scores were compared between age groups using one-way ANOVA tests, if normality and homogeneity assumptions were met, or Welch test when these assumptions were not met.

## 3 Results

Our sample was composed of 80 % of men and mean age was 38.4 (SD = 17.1). As for the age groups, 19.6 % were 18–21 y, 22.7 % were 22–29 y, 34.0 % were 30–49 y and 23.7 % were ≥ 50 y (Table 1). At the time of the intervention, 42.3 % were not working (29.3 % unemployed, 22.0 % retired and 46.3 % studying). On average, weekly drinking consumption was 13.4 standard units (SD=10.2) and AUDIT score was 9.53 (SD = 4.50) at baseline. When comparing age groups there were no significant differences in terms of gender, AUDIT score, and weekly drinking consumption at baseline (Table 1). There was a significant difference regarding occupational status (*Χ*^2^ (3, N = 97) = 20.97, *p* < .001, φ = .47), where the 18–21 y were mostly not working, i.e., 57.9 % studying and 15.8 % unemployed, and the 30–49 y group were mostly employed, i.e., 84.8 % employed.

**Table 1:** Baseline sociodemographic characteristics and alcohol use measures of the sample and by age group.

	N = 97	18–21y	22–29y	30–49y	≥ 50y	*p*
		19 (19.6)	22 (22.7)	33 (34.0)	23 (23.7)	
Male (N,%)	78 (80.4)	14 (73.7)	18 (81.8)	27 (81.8)	19 (82.6)	.89
Occupation (N,%)						**<.001**^**1**^
Not working	41 (42.3)	15 (78.9)	11(50.0)	5 (15.2)	10 (43.5)	
Student	19 (46.3)	11 (57.9)	8 (36.4)	0 (0.0)	0 (0.0)	
Unemployed	12 (29.3)	3 (15.8)	3 (13.6)	5 (15.2)	1 (4.3)	
Retired	9 (22.0)	0 (0.0)	0 (0.0)	0 (0.0)	9 (39.1)	
Other	1 (2.4)	1 (5.3)	0 (0.0)	0 (0.0)	0 (0.0)	
Working	56 (57.7)	4 (21.1)	11 (50.0)	28 (84.8)	13 (56.5)	
Baseline AUDIT score (M, SD)	9.5 (4.5)	11.3 (5.6)	9.7 (3.4)	9.6 (4.4)	8.0 (4.3)	.13
Baseline weekly drinking consumption, SU (M, SD)	13.4 (10.2)	9.5 (7.8)	13.2 (11.4)	14.9 (11.2)	14.5 (9.0)	.29

*Note*: ^1^ – *p* value of the-*X*^2^ test of independence between occupational status categories (working and not working) and age groups.

AUDIT, alcohol use disorder identification test; M, mean; SD, standard deviation; SU, standard units of alcohol.

When testing the influence of age on weekly drinking consumption, the negative binomial regression controlling for drinking at baseline showed that when compared with 22–29 y group (reference group), the 30–49 y group and the ≥ 50 y had significant differences. Specifically, for 30–49 y the predicted weekly drinking consumption was 60 % higher (IRR = 1.60, 95 % CI 1.02–1.10, *p* = .04) and 67 % for older adults (IRR = 1.67, 95 % CI 1.03–2.66, *p* = .03), compared with the reference group (Table 2). As for the change to low-risk drinking (Table 3), the logistic regression model showed that the 18–21 y group has 11.3 times the odds to remain at high risk of the reference group of 22–29 y (OR = 11.25, 95 % CI 1.19–106.7, *p* = .04).

**Table 2:** Negative binominal regression predicting weekly drinking consumption at follow-up.

				95 % CI	
	**IRR**	**SE**	***p***	**Lower Bound**	**Upper Bound**
22–29 y	*Reference*				
18–21 y	1.39	0.36	.20	0.84	2.32
30–49 y	1.60	0.37	.04	1.02	2.48
≥ 50 y	1.67	0.40	.04	1.03	2.66
Weekly drinking consumption at baseline	1.04	0.01	< .001	1.02	1.80
Gender	0.71	0.16	.12	0.47	1.09
AUDIT score	1.02	0.02	.44	0.97	1.06

*Note*: Female coded as 1 and male 0. AUDIT, alcohol use disorder identification test; B, unstandardized coefficients; CI, confidence interval; IRR, incidence rate ratio; SE, standard error. Test statistics: *Χ*^2^ (6, N = 97) = 41.8, *p* < .001

**Table 3:** Logistic regression predicting change to low-risk drinking at follow-up.

				95 % CI	
	**OR**	**SE**	***p***	**Lower Bound**	**Lower Bound**
22–29 y	*Reference*				
18–21 y	11.2	12.9	.04	1.19	106.7
30–49 y	2.89	1.91	.11	0.79	10.49
≥ 50 y	1.68	1.14	.45	0.44	6.62
Gender	0.51	0.33	.30	0.14	1.80
AUDIT scores	1.15	0.09	.09	0.98	1.35

*Note*: Female coded as 1 and male 0. AUDIT, alcohol use disorder identification test; OR, Odds Ratio; CI, confidence interval; SE, standard error. Test statistics: *Χ*^2^ (5, N = 97) = 13.2, *p* = .02

**Fig. 2:**
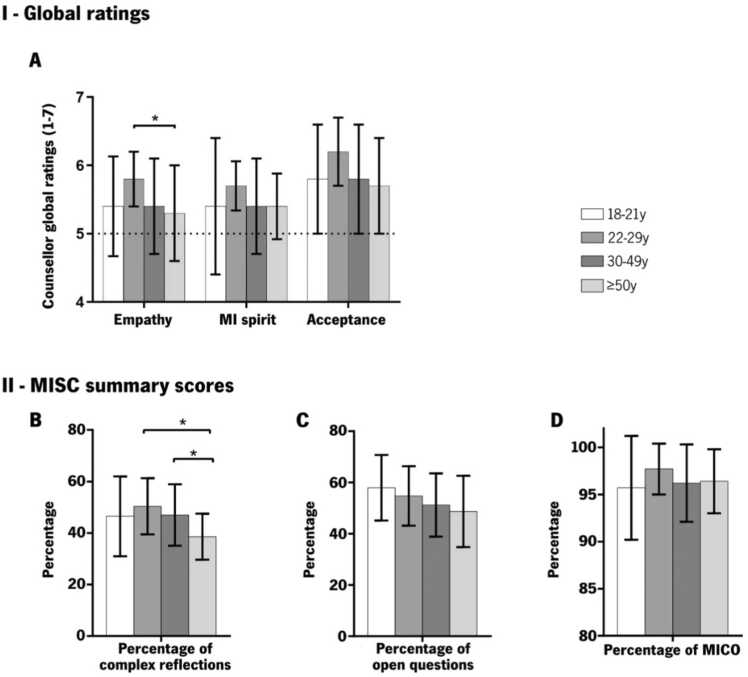
*Counsellor Global Ratings and MISC Summary scores by age group*. Higher counsellor global ratings (**I**) and MISC summary scores (**II**) for the 22–29 y group (except for the percentage of open questions): (**A**) empathy (F(3, 93) = 2.70, *p* = .05), MI spirit F_welch_(3, 46.9) = 2.04, acceptance (F(3, 93) = 1.70, *p* = .17), (**B**) percentage of complex reflections: F(3, 93) = 4.10, *p* = .009; (**C**) Percentage of open questions: F(3, 93) = 2.16, *p* = .10; (**D**) Percentage of MICO: F_welch_(3, 47.1) = 1.43, *p* = .25. MISC, motivational interviewing skill code, MICO, MI-consistent responses, *  denotes *p* < .05.

When examining MI counsellor skills by age group, a few differences between age groups were observed even if counsellors displayed high global ratings overall (Fig. 2). The age group 22–29 y displayed higher scores across all counsellor global ratings followed by 18–21 y and 30–49 y and with the ≥ 50 y displaying the lowest ratings. Specifically, the 22y-29y group sessions were coded as having 7.4 % higher counsellor empathy than the 18–21 y and 30–49 y groups and 9.4 % higher than the older group [F(3, 93) = 2.7, *p* = .05] (Fig. 2.A). When compared with the oldest group, the percentage of complex reflections was 30.6 % higher in the 22–29 y (*p* = .006) and 21.8 % higher in the 30–49 y (*p* = .048) (Fig. 2.B). The percentage of open questions tended to decrease when age increased (Fig. 2.C), but the age groups did not statistically differ (*p* = .10). The 22–29 y group displayed higher percentages of MICO (Fig. 2.D) but the age groups did not statistically differ (*p* = .25).

## 4 Discussion

In this exploratory study, we tested whether the outcomes of a BMI aiming at decreasing alcohol use among ER patients screened as having unhealthy alcohol use varied according to patients’ age group and whether this was associated with counsellors’ MI skills. Findings showed that the 22–29 y group had the best alcohol outcomes across both measures. They recorded statistically lower weekly drinking consumption at follow-up (controlling for baseline), compared to the 30–49 y and ≥ 50 y group. They were also more likely to change to low-risk drinking at follow-up, when compared with the 18–21 y group. Moreover, our findings showed that counsellors’ MI skills during the BMI sessions of the 22–29 y group were higher overall (i.e., higher counsellor global ratings and complex reflections than the other age groups). On the other hand, MI-related mechanisms were in general the lowest among older adults ≥ 50 y.

In our analysis, patients aged 18–21 y were more likely to remain in unhealthy consumption at follow-up as compared to those aged 22–29 y. Combining these two age groups might partly explain the small effect sizes reported among young adults [e.g. standardized mean difference of −0.11 when examining quantity of alcohol consumed among young adults aged up to 25 years (Foxcroft et al., 2016); g = 0.17 in terms of alcohol consumption among young adults aged 19–30 y (Tanner-Smith and Lipsey, 2015)]. Another explanation for these results might be the timing of the follow-up. Larger effect sizes were reported at shorter follow-up (<4 months) among individuals aged up to 25 years (Foxcroft et al., 2016). In the present study, there was no short-term assessment, which might have hidden short-term effects in some age groups. Similarly, it is also possible that a longer and/or more frequent sessions would be required to sustain the efficacy of the intervention in these groups (Tanner-Smith and Lipsey, 2015).

The secondary aim of this paper was to explore MI skills by age groups. The coding analysis showed that the young adults’ group, i.e., 22–29 y, displayed higher counsellors’ empathy ratings, and higher percentage of complex reflections. Recent studies have found associations between MI skills and CT and/or behavior change. For instance, global ratings such as MI spirit and empathy, when studied as a latent construct, were positively associated with reflections of CT and ST, which in turn predicted CT (Villarosa-Hurlocker et al., 2019). Interestingly, complex reflections have been positively associated with client preparatory talk at lag 1, i.e., a complex reflection was immediately followed by client preparatory talk, and predictive of strong client commitment talk at lag 2, i.e., the second event after a complex reflection (Brown et al., 2018). Additionally, the proportion of complex reflections was associated with higher proportion of CT, and higher proportion of CT was related to better behavior change outcomes (Magill et al., 2018). These effects might explain the associations between MI-related parameters and better alcohol outcomes at follow-up.

However, it remains unclear why this association was only observed in the 22–29 y age group. Thus, the next step would be to consider why the young adults group differed from the other age groups in terms of MI-related summary scores. One explanation could be the age of the counsellors, who were also young adults between 22 and 29 y. As the counsellors' and patients' ages matched, it is possible that the counsellors could better relate to the experiences and ambivalence of the 22–29 y group than to the other age groups and subsequently better work with them, leading to better outcomes in the end. In MI theory, empathy is defined as the extent to which a counsellor communicates accurate understanding of the patient’s views and experiences and is mirrored by reflections (Miller and Rollnick, 2013). Empathy might be put into practice using complex reflections, i.e., reflections that add additional meaning beyond what the client said, which aim at deepening the understanding by clarifying whether the counsellor's guess was accurate (Miller and Rollnick, 2013). Interestingly, this participant age group displayed the highest global ratings of counsellor empathy and a significantly higher proportion of complex reflections, which lends weight to the hypothesis of the importance of age in behavior change interventions.

Besides the possible contribution of the counsellor's age, it may well be the case that the nature of the empathic process is affected by the age of the patients. Whereas according to MI empirical theory empathy relates to how well the counsellor understands patient's perspectives and communicates this understanding, other authors have posited that empathy goes beyond the counsellor's response. For instance, Barrett-Lennard (1981) argued that empathy also comprises the patient's response to the counsellor's communication. This means that empathy is an interactive process (Finset and Ørnes, 2017) and the way the patients respond to the counsellor's communication might contribute to how the session unfolds. In fact, MI research that examined empathy and language style synchrony, i.e., the occurrence of both counsellor and patient use of the linguistic word count categories, found that high empathy sessions showed greater language style synchrony when compared with low empathy sessions (Lord et al., 2015). Thus, as growing evidence indicates that older adults display lower cognitive empathy, i.e., the ability to understand others' thoughts and feelings (Beadle and De La Vega, 2019), this interactive process might be hindered in this participant age group. This could manifest as a reduced patient’s ability to appreciate doctor’s perspective and intentions which in turn might lead to increased difficulty in expressing concerns. Consequently, this may make harder for the health professional to gather critical information, potentially contributing to lower values of the MI-related mechanisms as opposed to younger age groups. Further studies should explore these potential mechanisms and interactions, along with other factors such as cognitive status and aspects that typically matter to patients during their interactions with healthcare professionals including but not limited to perceived warmth, perceived level of dedication, and interactions with computers or other devices during the appointment (Marcinowicz et al., 2014, Pratiwi et al., 2023).”

As with all studies, there are limitations that offer opportunities for further research. First, the small sample size precludes a more detailed study of the factors that might moderate the observed effects. The small sample size along with the fact that only a sub-sample of the intervention group was recorded and coded, limit the generalization of the results (even if previous study of the same data showed no significant differences between coded and non-coded sessions) (Gaume et al., 2007). Second, the setting of the study should be considered and our findings might be limited to similar populations, e.g. non-treatment seekers, ER patients. Yet, this is also the situation in real clinical practice where health professionals are faced with patients that would benefit from a behavior change without necessarily acknowledging it nor expecting a targeted intervention. Conversely, besides the study design, one of the main strengths of this study was the structured BMI model that allowed to compare age groups while ensuring that many aspects of the intervention are standardized, and thus minimize between-session variability.

## 5 Conclusions

In summary, our study shows that a single BMI provided within an ER setting was associated with significantly better 12-month alcohol outcomes among patients aged 22–29 years, and that this effect was associated with higher MI skills during the session among this age group (higher counsellor empathy ratings and higher percentage complex reflections), as compared with other age groups. Altogether, our findings suggest that patient's age might influence the effectiveness of MI. This highlights aspects so far scarcely explored in this field, such as the impact of patient's age or the match between the counsellor's and patient's age, that may account for the mixed results that have been reported for BMI in the ER (Daeppen et al., 2007, Landy et al., 2016, Barata et al., 2017). We argue that patients' age by itself or in interaction with the counsellors' age might partly explain both the differences found in terms of how the counsellors apply the MI principles and in turn the effectiveness of BMI. Future studies should confirm these findings and further explore which adjustments health professionals can put into practice to respond to this challenge.

## Role of funding source

This work was supported by Swiss Government (Swiss Government Excellence Research Scholarship 2019/2020, Belina Rodrigues) and the parent trial (Daeppen et al., 2007) was funded by Grant # 3200-067949 of the Swiss National Science Foundation.

## CRediT authorship contribution statement

**Jacques Gaume:** Writing – review & editing, Supervision, Project administration, Methodology, Data curation, Conceptualization. **Jean-Bernard Daeppen:** Writing – review & editing, Resources, Project administration, Methodology, Investigation, Funding acquisition, Conceptualization. **Nicolas Bertholet:** Writing – review & editing, Supervision, Project administration, Methodology, Data curation, Conceptualization. **Belina Rodrigues:** Writing – review & editing, Writing – original draft, Visualization, Methodology, Formal analysis, Data curation.

## Declaration of Competing Interest

none.
